# Thumb Imprint Based Detection of Hyperbilirubinemia Using Luminescent Gold Nanoclusters

**DOI:** 10.1038/srep39005

**Published:** 2016-12-15

**Authors:** Srestha Basu, Amaresh Kumar Sahoo, Anumita Paul, Arun Chattopadhyay

**Affiliations:** 1 Department of Chemistry, Indian Institute of Technology Guwahati, Guwahati 781039, India; 2 Centre for Nanotechnology, Indian Institute of Technology Guwahati, Guwahati 781039, India

## Abstract

Early and easy detection of diseases, using point-of-care and inexpensive devices, not only provides option for early treatment but also reduces the risk of propagation. Herein we report the fabrication of a robust film based luminescence indicator of bilirubin, which can indicate hyperbilirubinemia through the thumb imprint of the patient. The UV-light induced luminescence intensity of the film, made out of chitosan stabilised gold (Au) nanoclusters, which was effectively quenched in the presence of Cu^2+^ ions, recovered in the presence of bilirubin from skin or blood serum. Moreover, the sensitivity of detection of bilirubin was tuneable with the amount of Cu^2+^ added, thereby facilitating the detection of the desired concentration range of bilirubin.

The significant increase in global population - in a rather short period of time - demand addressing persistent healthcare issues of a large populace. The current stress on the development of point-of-care diagnostics, especially important for remote and resource-deficient regions, is engendering the development of easy to use devices^1^. Among the widely occurring disease conditions, hyperbilirubinemia, which is prevalent among the newborns and jaundice afflicted patients, is a health condition where bilirubin concentration in blood plasma crosses 1.19 × 10^−2^ mg/mL in adults and 19.8 mg/mL in the newborns. Bilirubin, produced from the catabolism of hemoglobin present in the red blood cells, is excreted through bile and urine. The disease is generally correlated with liver disfunction or biliary obstruction; however, hemolytic anemia can also lead to production of excess bilirubin. It is also associated with jaundice leading to yellowing of skin color. Typically, hyperbilirubinemia is identified in the sclera (protective outer layer of the eye) by visual observation, urine, stool and other mucous membranes with the appearance of yellow color^2^. Early detection and treatment hold the keys to preventing the condition reaching to critical level. These call for the use of the latest of science and technology for the development of the smartest of devices useful for common people at large.

In this regard, nanoscale science and technology - in combination with chemistry-based technology - provide ingenious options in probing health conditions at the highest possible sensitivity and at the fastest speed. For example, conventional pathological laboratory tests use biochemical reactions to provide information - among a plethora of other molecular markers - about total sugar, cholesterol, triglycerides and uric acid. These are generally based on optical markers with absorption in the visible region of wavelengths being the primary indicator. Moreover, research efforts over the past decades have led to significant advancements in disease detection using microRNAs as biomarkers. With regard to cancer detection, microRNA based markers have been employed for accurate prognosis of malignancy^3^. Further, “amplification free” counting of microRNAs has been made possible by a kinetic fingerprint based approach, which surpasses conventional techniques in terms of specificity and identifying false positive results^4^. Recent reports also lay stress on developing biosensors based on principle of “mechanochemical” sensing of analytes, which allow sensitive detection at single molecule level^5^. On the other hand, nanoscale particles such as metal nanoparticles, quantum dots, carbon dots, graphene quantum dots and atomic clusters usually provide higher sensitivity to detection using either surface plasmon resonance based optical changes or changes in the emission characteristics of the nanoparticle based sensor^6^,^7^,^8^. Thus, through observation of changes in their characteristics optical properties, identification of specific DNA and RNA sequences, proteins, toxins, bacteria and viruses have been possible^9^,^10^,^11^. In contrast, luminescent nanomaterials mentioned above offer sensitive detection of pathogens, important biomolecules and narcotics and act as molecular markers of DNA and protein based diseases^12^,^13^. The large Stokes-shifted and tunable emission of the materials provide additional advantage of sensing molecules even at the femto-molar scale. As regards to hyperbilirubinemia, a recent report involving luminescence quenching in the presence of bilirubin indicated potential for use of Au nanoclusters for such sensing^14^. However, selectivity, specificity and potential device fabrication, which were not addressed in the report, are essential for the development of a practicable sensor. This is where the principle of chemistry could be employed in conjunction with the versatility of nanomaterials.

Herein we report the fabrication of a new nanotechnology-based luminescent sensor for hyperbilirubinemia. The device is a film made of chitosan-stabilized Au nanoclusters embedded in a polyvinylidene difluoride (PVDF) membrane. The said device was capable of detecting bilirubin with high sensitivity through thumb impression or from the blood plasma of a jaundice afflicted patient. In addition, we report a luminescence based detection of blood bilirubin with high sensitivity in the liquid phase. The film appeared luminescent yellow in the presence of UV light. The luminescence intensity was quenched in the presence of copper salt such as copper sulphate. On the other hand, in the presence of bilirubin, the intensity was restored.

## Results and Discussions

**Figure 1:**
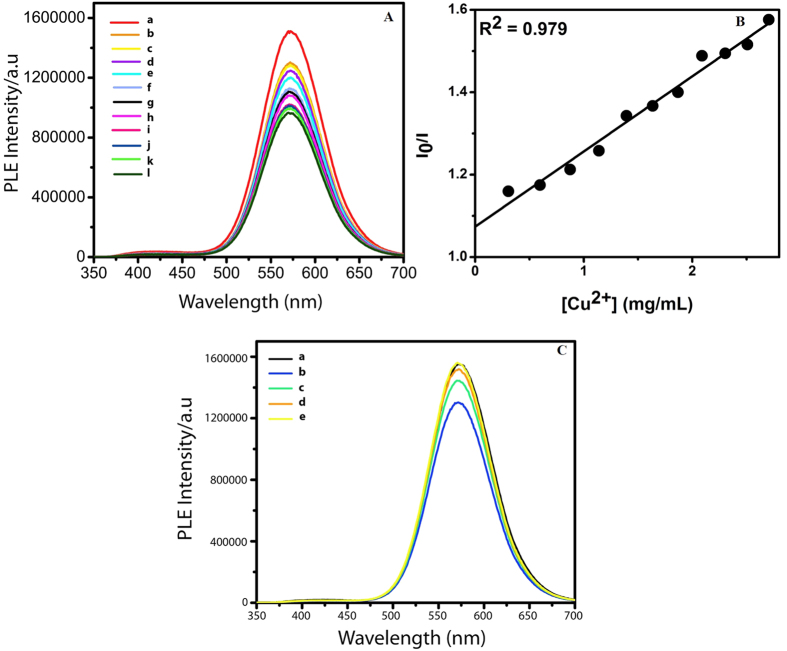
(**A**) Gradual quenching of luminescence intensity of Au nanoclusters upon gradual addition of Cu^2+^. Photoluminescence emission (PLE) spectrum of (a) Au nanoclusters and of that following addition of (b) 3.058 × 10^−1^ mg/mL, (c) 5.971 × 10^−1^ mg/mL, (d) 8.748 × 10^−1^ mg/mL, (e) 1.14 mg/mL, (f) 1.393 mg/mL, (g) 1.635 mg/mL, (h) 1.867 mg/mL, (i) 2.090 mg/mL, (j) 2.303 mg/mL, (k) 2.505 mg/mL and (l) 2.704 mg/mL Cu^2+^ salt solution. (**B**) Corresponding Stern-Volmer plot. I_0_ is the luminescence intensity of as-synthesized Au nanoclusters and I is the reduced luminescence intensity of the same on subsequent addition of Cu^2+^ ions. (**C**) The figure shows luminescence intensity quenching of Au nanoclusters upon addition of Cu^2+^ and recovery of the same on addition of BR (bilirubin). PLE spectrum of (a) Au nanoclusters and of that following addition of (b) 1.38 mg/mL Cu^2+^ and then (c) 5.8 × 10^−4^ mg/mL BR, (d) 1.1 × 10^−3^ mg/mL BR and (e) 1.5 × 10^−3^ mg/mL BR, respectively. BR means bilirubin.

**Figure 2:**
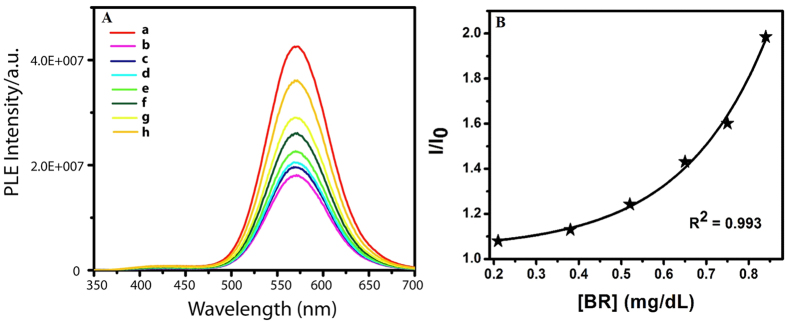
(**A**) Graphs showing gradual luminescence intensity recovery of Au nanoclusters otherwise quenched in the presence of Cu^2+^ ions. PLE spectrum of (a) Au nanocluster dispersion and of that following addition of (b) 4.77 mg/mL Cu^2+^, (c) 2.1 × 10^−3^ mg/mL BR, (d) 3.8 × 10^−3^ mg/mL, (e) 5.2 × 10^−3^ mg/mL BR, (f) 6.5 × 10^−3^ mg/mL BR, (g) 7.5 × 10^−3^ mg/mL BR and (h) 8.4 × 10^−3^ mg/mL BR, respectively. (**B**) Gradual increase of normalized luminescence intensity of the Cu^2+^ quenched Au nanocluster dispersion after addition of bilirubin. I_0_ is the reduced luminescence intensity of the nanoclusters in the presence of copper ions. I is the recovered luminescence intensity on subsequent addition of BR. BR means bilirubin.

Experimentally, luminescent Au nanoclusters in chitosan were synthesized by modifying a protocol established in our laboratory^15^ (Supplementary Fig. S1A,B). Gradual addition of copper sulphate solution (12.5 mg/mL) to the 0.8 mL aqueous dispersion of the clusters (0.1 mL Au nanoclusters dispersion and 0.7 mL glycine buffer) led to lowering of intensity of luminescence^16^ (Fig. 1A). The pH of the medium was maintained at 2.5 using glycine buffer (Supplementary Information), which was required for subsequent experiment as bilirubin is known to quench the luminescence of Au nanoclusters at pH above 2.59 (Supplementary Fig. S2). Corresponding Stern-Volmer constant was calculated to be (1.65 ± 0.21) × 10^−3^ M^−1^ (Fig. 1B). It was further observed that addition of bilirubin to a system comprising of Cu^2+^ added Au nanoclusters (the luminescence of which was quenched due to the presence of Cu^2+^ ions) led to the recovery of the luminescence. Thus when a solution containing 1.38 mg/mL of copper ions in 0.8 mL Au nanocluster dispersion was subsequently treated with increasing amount of bilirubin, the intensity of luminescence increased systematically up to full recovery (Fig. 1C). Control experiment involving addition of water (instead of bilirubin solution) did not lead to significant change in the luminescence intensity (Supplementary Fig. S3). Further bilirubin did not seem to have any effect on luminescence intensity of the clusters in the absence of Cu^2+^ions (Supplementary Fig. S4A,B). Also, a mixture of Cu^2+^(12.10 mg/mL) and bilirubin (5 × 10^−4^ mg/mL), when added to Au nanocluster dispersion, did not lead to significant change in luminescence intensity when compared to the effect of sequential addition of copper ions and then bilirubin (Supplementary Fig. S5). The results suggested important role of bilirubin in the restoration of Au nanocluster luminescence even in the presence of Cu^2+^ ions. Similar experiments were performed with different concentrations of copper ions and bilirubin (Fig. 2A). Corresponding plots of recovery of luminescence versus concentrations of adducts (akin to Stern-Volmer plot) were obtained (Fig. 2B). The purpose of the experiments was to find the lowest concentration of bilirubin, which would ensure the observation of change of luminescence with the highest sensitivity. It was found that bilirubin concentration as low as 8.4 × 10^−3^ mg/mL could provide detectable change in the luminescence intensity of Au nanoclusters (through recovery) in the presence of Cu^2+^ ions, the concentration of which could be as high as that of 4.77 mg/mL. On a similar note, blood serum of a jaundice afflicted patient with bilirubin concentration of 6.4 × 10^−2^ mg/mL was diluted a thousand times and added to Au nanoclusters dispersion with lower Cu^2+^ concentration (1.38 × 10^−2^ mg/mL). This also resulted in significant luminescence recovery. Thus, it was possible to detect varying amount of bilirubin just by tuning the amount of Cu^2+^ ions (Supplementary Table S1). The recovery of luminescence intensity of the nanoclusters by bilirubin in the presence of 4.77 mg/mL Cu^2+^ ions could be fitted with single exponential function. The results showed that use of higher concentration of Cu^2+^ ions in the medium helped detect higher amounts of bilirubin.

Time-resolved photoluminescence measurements indicated average luminescence lifetime of as-synthesized Au nanoclusters to be 1.56 μs, which decreased to 1.29 μs upon addition of 0.2 mL of Cu^2+^ (5 × 10^−4^ M) and subsequently was restored to 1.53 μs upon further addition of bilirubin (from blood serum with bilirubin concentration of 6.4 × 10^−5^ mg/mL) (Supplementary Fig. S6). The quenching of luminescence of Au nanoclusters by Cu^2+^ ions is likely to be dynamic in nature. This is apparent from the fact that in addition to the linear nature of the Stern-Volmer plot for quenching of luminescence of Au nanoclusters by Cu^2+^ ions, significant changes in the luminescence lifetime of the nanoclusters in the presence of Cu^2+^ was observed. Interestingly, UV-vis spectrum indicated the appearance of a peak at 350 nm owing to the Cu-bilirubin complex^17^,^18^, which was neither present in the sample containing Au nanoclusters nor in Cu^2+^ ion added Au nanoclusters (Supplementary Fig. S7). Thus, overall, while Cu^2+^ addition led to quenching of luminescence due to Au nanoclusters, addition of bilirubin led to recovery of the same, possibly owing to the formation of Cu-bilirubin complex^17^,^18^.

Since the luminescence of the Au nanoclusters (embedded in chitosan) was responsive to the presence of Cu^2+^ ions and subsequently to bilirubin an opportunity was apparent with respect to making a device for the detection of bilirubin with high sensitivity. Also since bilirubin is known to form complex with Cu^2+^ having known stoichiometry^17^, the detection could have the advantage of being quantitative. Further, common techniques which involve loss or lowering of luminescence in the presence of an analyte may not necessarily lead to detection with high sensitivity. On the other hand, recovery of luminescence in the presence of an analyte may offer unique opportunity of sensing with enhanced sensitivity. This – in principle – could provide an eminent opportunity for making a solid-state device for the above purpose.

**Figure 3:**
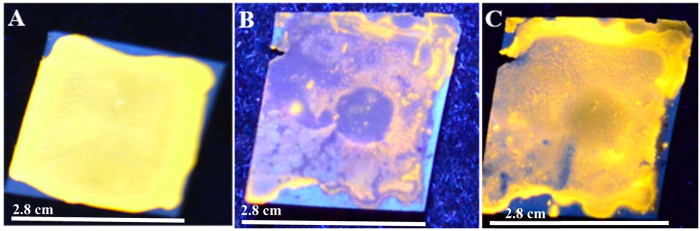
Photograph of (**A**) Au nanocluster coated polyvinylidene difluoride (PVDF) membrane (with dimension of the films being 2.8 × 2.8 cm^2^) as visualized under UV lamp with excitation at 254 nm. (**B**) Au nanocluster coated PVDF membrane as observed using 254 nm UV light, following addition of copper sulphate solution. The luminescence can be observed to have been reduced substantially. (**C**) BR (bilirubin) treated film as viewed using 254 nm UV light. The restoration of the intense yellow-orange color indicated recovery of luminescence of the film.

In order to pursue the above objective, the as-prepared Au nanoclusters were drop-cast on a PVDF membrane (which was cut in the form of a film of ~2.8 × 2.8 cm^2^ size) in which the bright yellow-orange luminescence of the clusters in the presence of UV light (254 nm) could easily be observed (Fig. 3A). Further, when the film was treated with CuSO_4_ solution (0.2 mL of 12.5 mg/mL) the luminescence disappeared nearly completely in the area where the liquid was dropped (Fig. 3B). On the other hand, when the same film was further treated with 0.2 mL of 1.1 × 10^−2^ mg/mL bilirubin the luminescence recovered with great clarity (Fig. 3C). Control experiment, involving addition of water to the copper treated (and Au nanoclusters coated) PVDF membrane did not show restoration of luminescence intensity, thus clearly indicating the role of bilirubin in the recovery (Supplementary Fig. S8). It is plausible that Cu^2+^ ions when present in the film (following addition of an aqueous solution of CuSO_4_) acted as the quencher of luminescence. However, when bilirubin solution was added to the film, Cu-bilirubin complex formation might have taken place. The complex might not have quenched the luminescence and thus the recovery of luminescence was achieved. The above results indicated the potency of a new device in the form of solid film for the detection of bilirubin present in the aqueous medium.

**Figure 4:**
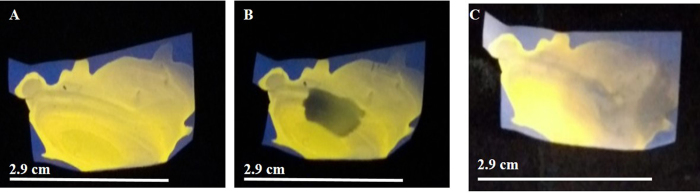
(**A**) Digital photograph of Au nanoclusters containing PVDF membrane (dimension of the films was 2.9 × 2.1 cm^2^). (**B**) Copper salt (20 μL 12.4 mg/mL) treated Au nanoclusters containing PVDF membrane. The low luminescence region is due to quenching by Cu^2+^ ions added to the membrane. (**C**) The same film after thumb impression of a jaundice afflicted patient. The photographs were recorded following illumination with UV light (254 nm).

As discussed above, bilirubin is known to be present in the skin of a person affected with hyperbilirubinemia. The fact that deposition of bilirubin occurs on skin of a jaundice afflicted patient, has been widely used as a tool to correlate serum bilirubin concentration. In this regard, various studies, including detailed theoretical study, involving mechanism of transfer of bilirubin from plasma to skin have been reported^19^,^20^,^21^. Thus, probing the molecular signature of a disease – for example hyperbilirubinemia in the present study- through chemical reaction with another species, thereby leading to prominent optical changes, could emerge as a new avenue for disease diagnosis. Hence it was deemed feasible to test the efficacy of the device in detecting the presence of excess bilirubin through the thumb imprint of an affected patient. In order to test this a film (as above) was fabricated consisting of Au nanoclusters (embedded in chitosan) in PDVF membrane. The luminescence of the film was recorded using UV light as the excitation source (Fig. 4A). The film was then treated with 0.02 mL of 12.5 mg/mL CuSO_4_ solution upon which the luminescence disappeared (Fig. 4B). Interestingly, when the thumb of a patient afflicted with hyperbilirubinemia was pressed against the copper-treated film for 5 min, the recovery of luminescence of the film could be observed (Fig. 4C). Pathological test in the laboratory indicated the concentration of bilirubin in the patient’s blood sample to be 3.1 × 10^−2^ mg/mL, which was well above the limit of concentration of a healthy adult. This experimental result has been presented thrice to support the repeatability of the results. In an analogous set of experiment, involving thumb imprint of a jaundice afflicted patient with bilirubin level recorded as 4.1 × 10^−2^ mg/mL, the quenched luminescence intensity of copper treated Au nanocluster film showed restoration of luminescence intensity when thumb imprint of the said patient was acquired on the film (Supplementary Fig. S9). On the other hand, when the same patient, immediately following the first impression, pressed the same thumb on another (similar) film of Au nanoclusters added with Cu^2+^ ions, no significant fluorescence recovery was obtained (Supplementary Fig. S10A–C). This could be due to insufficient bilirubin deposition on the patient’s skin following the first impression. Control experiment with healthy volunteer did not lead to any recovery of the luminescence of the copper-treated film (Supplementary Fig. S11). Similar results (for patients with bilirubin level within and above normal range) were obtained when chitosan stabilized Au nanoclusters were dried as film instead of coating on to PVDF membrane (Supplementary Fig. S12). In an allied vein, detection of bilirubin in blood serum of a jaundice afflicted patient (total bilirubin count of 6.4 × 10^−2^ mg/mL) was also possible using the liquid phase method (Supplementary Fig. S13).

## Conclusions

In essence, we have developed a new nanotechnology-based fast and easy method of identifying hyperbilirubinemia in patients afflicted with jaundice. The method invented provided an option to circumvent the need of blood test for fast analysis and used thumb impression instead for testing of hyperbilirubinemia. The method (and the device) could be considered cost-effective and versatile, as the analysis could be performed, in minutes, without the need of a typical pathological laboratory setup. The method involved the use of luminescent Au nanocluster film, the intensity of which was effectively quenched in the presence of copper salt. The detection method relied on the recovery of luminescence intensity in the presence of bilirubin. The change in intensity was equally effective in liquid medium as well as in the solid phase, thus providing a detection with high sensitivity. The facile detection of bilirubin was possible using blood serum as well as through thumb impression of the patient. The best sensitivity of detection of BR in the liquid phase was 6.4 × 10^−5^ mg/mL and that in the solid phase was achieved with the concentration of blood bilirubin in serum of the affected patient being ~3.1 × 10^−2^ mg/mL. The bilirubin detection technique developed herein is based on complexation reaction between bilirubin and copper ions and thus offers superiority in terms of selectivity over other conventional luminescence quenching based methods. However, disease markers capable of forming stronger complex with copper ions as compared to bilirubin might interfere in detection method developed herein. Also, significant alteration in pH of the system (beyond the buffer capacity of glycine buffer used herein) might lead to complexity in detection of hyperbilirubinemia by the said method as the luminescence of chitosan stabilized nanoclusters used here is itself sensitive to pH. However, for the solid device such an effect may not arise. In a nutshell, the solid device thus designed is superior to the commonly practiced method as it enabled prompt, easy and precise detection of jaundice and could be used as a point-of-care device especially by common mass in a resource-limited region.

## Materials and Methods

### Materials

Chitosan (Sigma Aldrich), tetra chloro auric acid (HAuCl_4_, Sigma Aldrich) mercaptopropionic acid (MPA, Sigma Aldrich), copper sulphate (Merck), bilirubin (BR, Alfa Aesar), glycine, sodium chloride and HCl were used as received. Milli-Q grade water was used for all experiments.

### Synthesis of luminescent Au nanoclusters

Au nanoclusters were prepared by adding 1.2 mL of aqueous solution of HAuCl_4_ (10 mM) to 20 mL of 0.5% (w/v) chitosan (which was solubilized using 0.1% glacial acetic acid) under stirring condition. This was followed by addition of 0.8 mL mercaptopropionic acid (MPA; 0.11 M). Stirring was continued for 30 min and the pH of the resulting medium was kept at or below 2.5 using 0.01 M HCl.

### Preparation of glycine buffer

58.0 mg of sodium chloride was added with 75.0 mg glycine in 10.0 mL water. The pH of the resulting medium was kept at 2.5 using 0.01 M HCl.

### Preparation of Au nanocluster dispersion for luminescence experiment

To 0.1 mL of Au nanocluster dispersion prepared using the above mentioned protocol (experimental section A), 0.7 mL of glycine buffer (experimental section C) was added. This was deemed essential to keep the absorbance value of the Au nanocluster dispersion below 0.1 in order to avoid self-absorption. Also, the pH of the medium was checked to ensure a value below 2.5. To this medium, required amount of copper sulphate and bilirubin solutions were added for further experiments.

### Preparation of bilirubin solution

Required amount of bilirubin was dissolved in appropriate amount of water and added with 1 μL of 1 M NaOH.

### Preparation of Au nanocluster containing film

The medium (30 mL) containing as-synthesized Au nanoclusters was poured onto a petri dish (Tarsons, disposable sterile petridish) and was then allowed to dry overnight in an oven at 55 °C. A film was formed with the approximate dimension of 3 × 3 cm^2^. The intrinsic luminescent properties of Au nanoclusters remained intact after the formation of the film. The film had yellow-orange luminescence upon excitation with 254 nm UV light.

In a similar way, the as-prepared Au nanocluster dispersion (1.0 mL) was drop-cast on an approximately 3 × 3 cm^2^ polyvinylidene difluoride (PVDF) membrane and the coated membrane –following drying - was used for further experiments. Since the films have been subjected to evaporation following drop casting of gold nanoclusters, the intensity of luminescence of gold nanoclusters (upon exposure to UV light) on the periphery of the film showed higher intensity owing possibly due to “coffee-ring effect”^22^.

### Treatment of the Au nanocluster containing film with copper sulphate

The Au nanoclusters coated polyvinylidene difluoride (PVDF) membrane (as above) was treated with 0.2 mL CuSO_4_ (12.5 mg/mL) solution. The film was then dried in air for 30 min and was observed under UV light. The luminescence intensity of the film got substantially reduced (upon exposure to 254 nm UV light) following addition of the copper salt.

### Interaction of bilirubin solution with copper sulphate added Au nanocluster containing film

0.2 mL aqueous solution of bilirubin (1.1 × 10^−2^ mg/mL) was added to Au nanocluster containing film, which was previously treated with 0.2 mL of CuSO_4_ (12.5 mg/mL) solution. The film was then dried for 30 min and then was viewed using UV light. Recovery of the yellow-orange luminescence of the film was observed.

### Interaction of copper salt with luminescent Au nanoclusters in liquid phase

To the above prepared dispersion of Au nanoclusters (experimental section E), varying concentration of copper sulphate solution was added sequentially for various experiments. The concentration for each of the solutions of copper sulphate is mentioned in the respective figure legends.

### Interaction of bilirubin with copper salt added luminescent Au nanoclusters in liquid phase

To Au nanocluster dispersion added with different amounts of copper salt, varying amount of bilirubin (both laboratory chemical and present in the blood serum) was added for various experiments, the concentration for each of which is mentioned in the figure legends.

### Control experiments

Control experiments including addition of water of equal amount as that of bilirubin solution to copper sulphate added Au nanoclusters, addition of bilirubin to pristine Au nanoclusters, addition of a mixture of copper-bilirubin mixture to Au nanoclusters, effect of thumb imprint of a volunteer with bilirubin concentration in normal range on copper salt treated film of Au nanoclusters, effect of thumb imprint of a jaundice afflicted patient on a film (copper treated Au nanoclusters) immediately following imprint on a similar film were performed.

### Collection of serum and thumb imprint of patients affected with hyperbilirubinemia

Serum and thumb imprints of jaundice patients were collected from hospital affiliated to Indian Institute of Technology Guwahati, India with full consent of the volunteers, following institutional protocol. Additional imprints were obtained from Guwahati Neurological Research Centre (GNRC) hospital, North Guwahati, Guwahati following appropriate procedure.

## Additional Information

**How to cite this article**: Basu, S. *et al*. Thumb Imprint Based Detection of Hyperbilirubinemia Using Luminescent Gold Nanoclusters. *Sci. Rep.* **6**, 39005; doi: 10.1038/srep39005 (2016).

**Publisher's note:** Springer Nature remains neutral with regard to jurisdictional claims in published maps and institutional affiliations.

## Supplementary Material

Supplementary Information
