# Supplementary material for: Thumb Imprint Based Detection of Hyperbilirubinemia Using Luminescent Gold Nanoclusters
Source: Sci Rep. 2016 Dec 15;6:39005. doi: 10.1038/srep39005 (PMC5157017; doi:10.1038/srep39005)
Supplement: Supplementary Information [file srep39005-s1.pdf]

Supplementary Information

Thumb Imprint Based Detection of Hyperbilirubinemia Using Luminescent Gold Nanoclusters

Srestha Basu[a], *Amaresh Kumar Sahoo*[b], *Anumita Paul**[a] *and Arun Chattopadhyay* *[ab]

[a]Department of Chemistry, Indian Insitute of Technology Guwahati, Guwahati 781039, India

[b]Centre for Nanotechnology, Indian Insitute of Technology Guwahati, Guwahati 781039, India

*Email: arun@iitg.ernet.in; anumita@iitg.ernet.in

Results

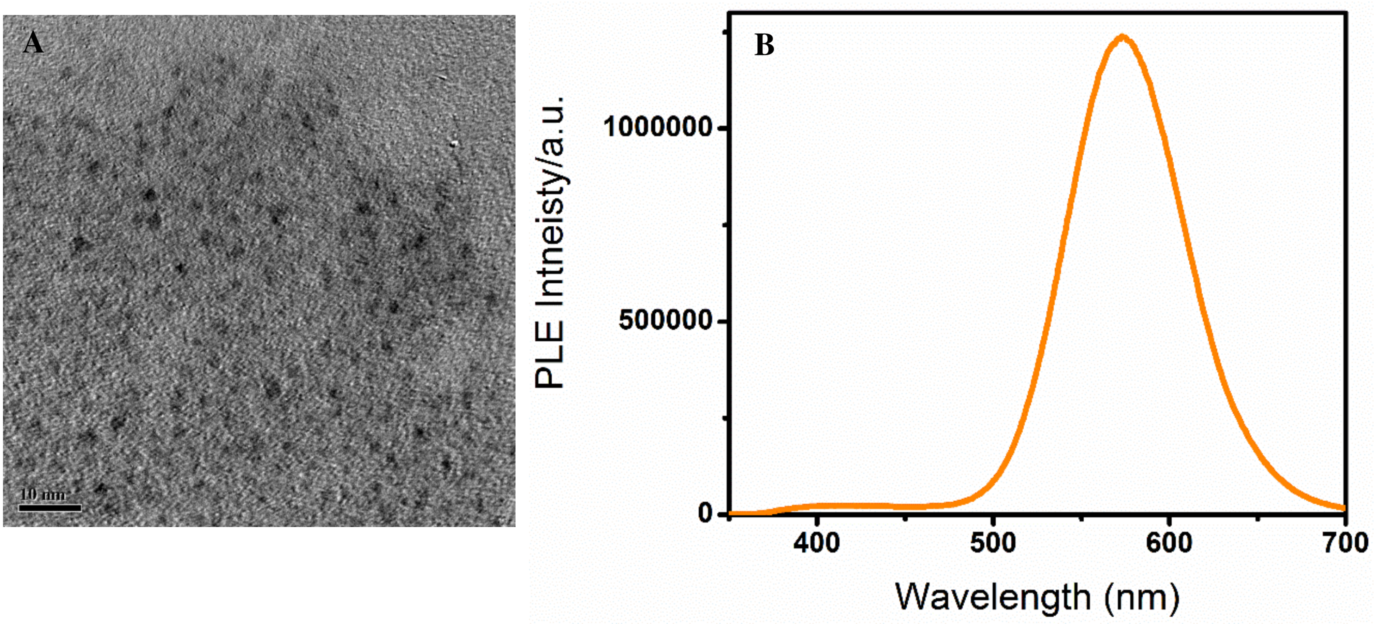

Supplementary Fig. S1. (A) TEM image of as-synthesized Au nanoclusters. (B) Luminescence emission spectrum of as-synthesized Au nanoclusters. The excitation wavelength was set at 300 nm.

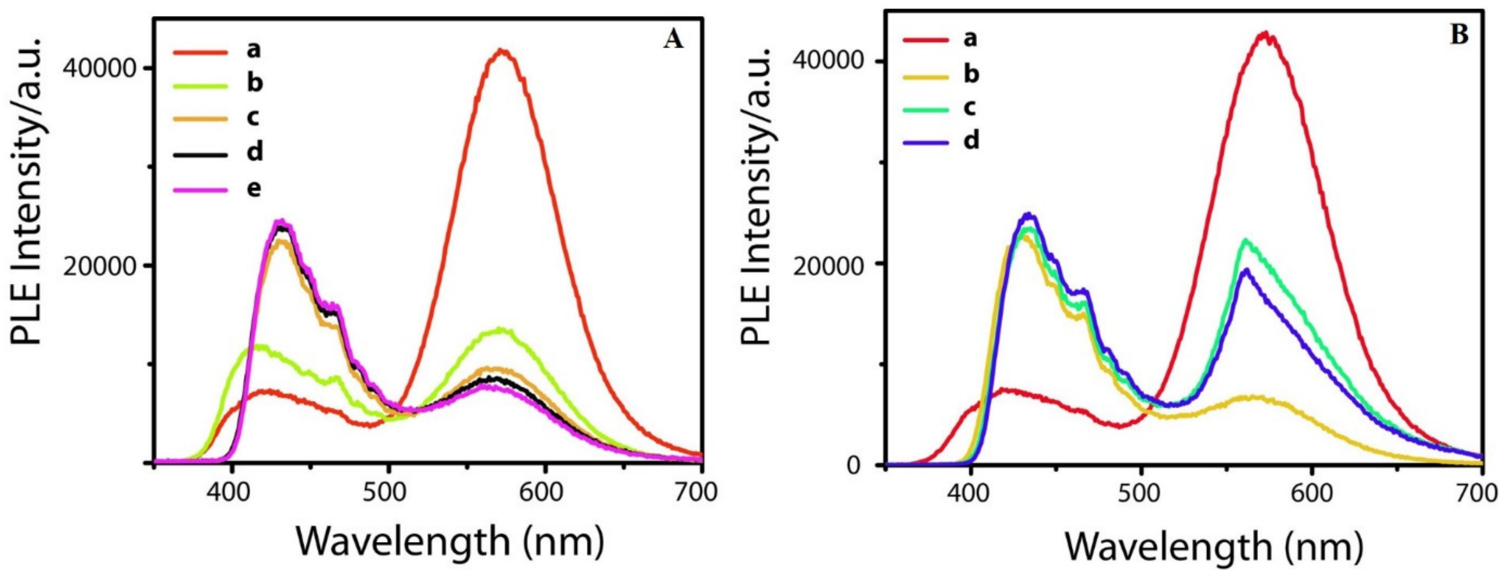

Supplementary Fig. S2. (A) Photoluminescence emission (PLE) spectra of (a) as-synthesised Au nanoclusters, (b) 10 µL Cu^{2+} (10 mg/mL) added Au nanoclusters, (c) 10 µL bilirubin (10×10^{-2} mg/mL) added Au nanoclusters; sample recorded after (d) 5 min and (e) 10 min of addition of the said amount of bilirubin. (B) Luminescence emission spectra of (a) as-synthesised Au nanoclusters, (b) 10 µL Cu^{2+}(10 mg/mL) added Au nanoclusters, (c) 10 µL bilirubin (10 mg/mL) added Au nanoclusters; sample recorded after (d) 10 min of addition of the said amount of bilirubin. The pH of Au nanocluster dispersion was maintained at 2.7 for all samples. In this particular experiment, sodium hydroxide was not used to solubilize bilirubin.

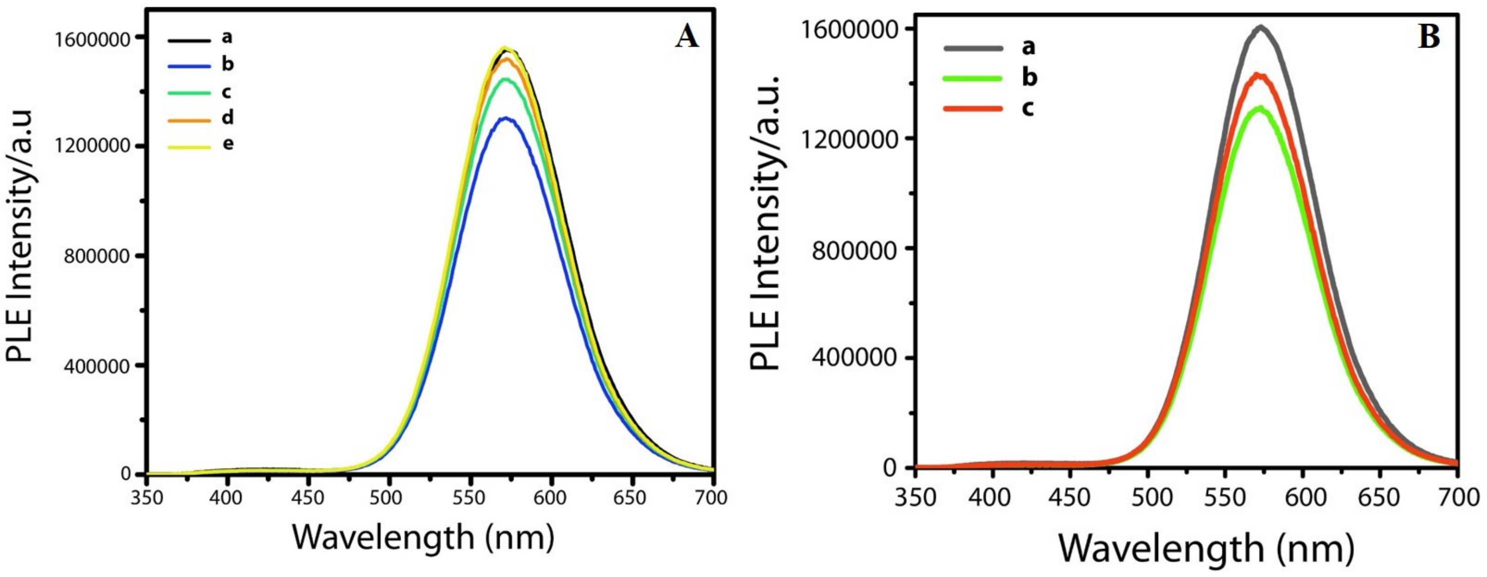

Supplementary Fig. S3. Effect of the same amount (volume) of (A) bilirubin (in water); PLE spectra of (a) Au nanoclusters following addition of (b) 1.38 mg/mL Cu^{2+}, (c) 5.8×10^{-4} mg/mL BR, (d) 1.1×10^{-3} mg/mL BR and (e) 1.5×10^{-3} mg/mL BR and (B) water addition on the luminescence intensity of copper added Au nanoclusters (Au NCs) dispersion; PLE spectra of (a) Au nanoclusters following addition of (b) 1.38 mg/mL Cu^{2+} and (c) 150 µL of water (the same volume of BR showing full recovery of luminescence intensity as shown in (A)). BR means bilirubin.

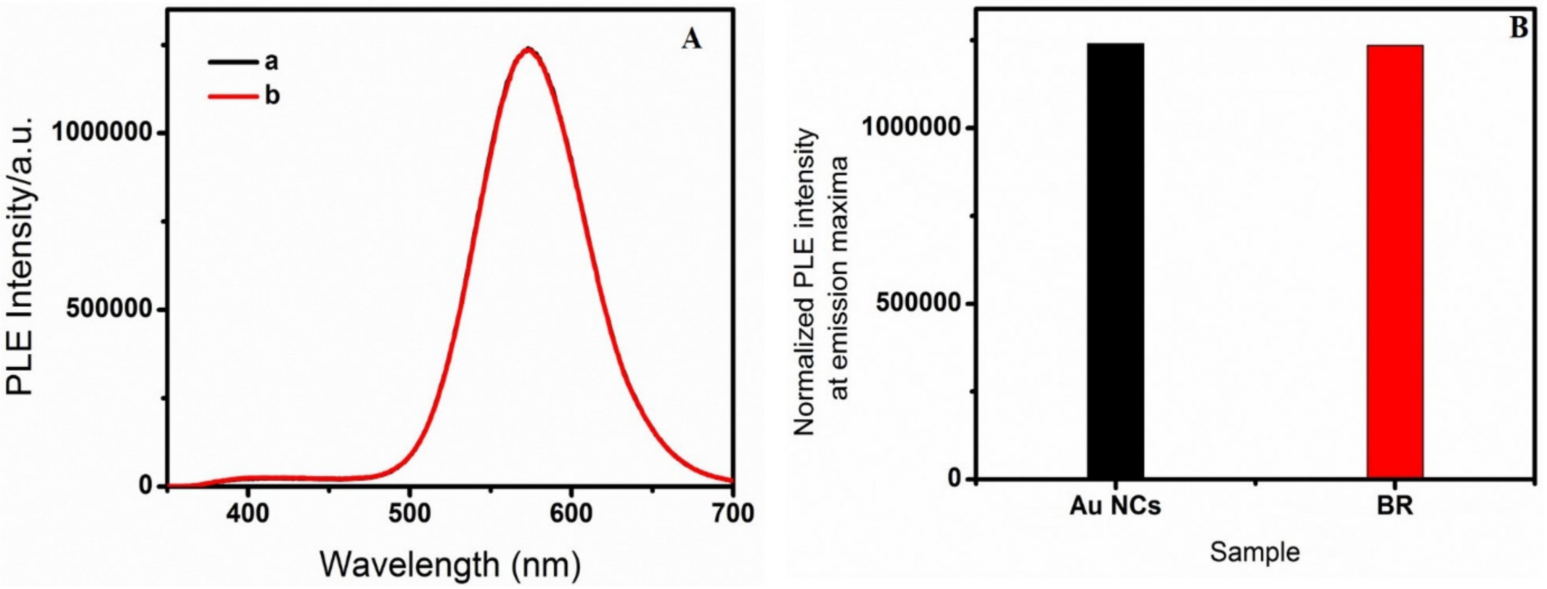

Supplementary Fig. S4 A- B. Effect of bilirubin (BR) on luminescence intensity of as-synthesized Au nanocluster (Au NC) dispersion. (A) Photoluminescence emission (PLE) spectrum of Au nanoclusters (a) before and (b) after addition of bilirubin. (B) Normalized PLE intensity of Au nanoclusters before and after addition of bilirubin at emission maxima.

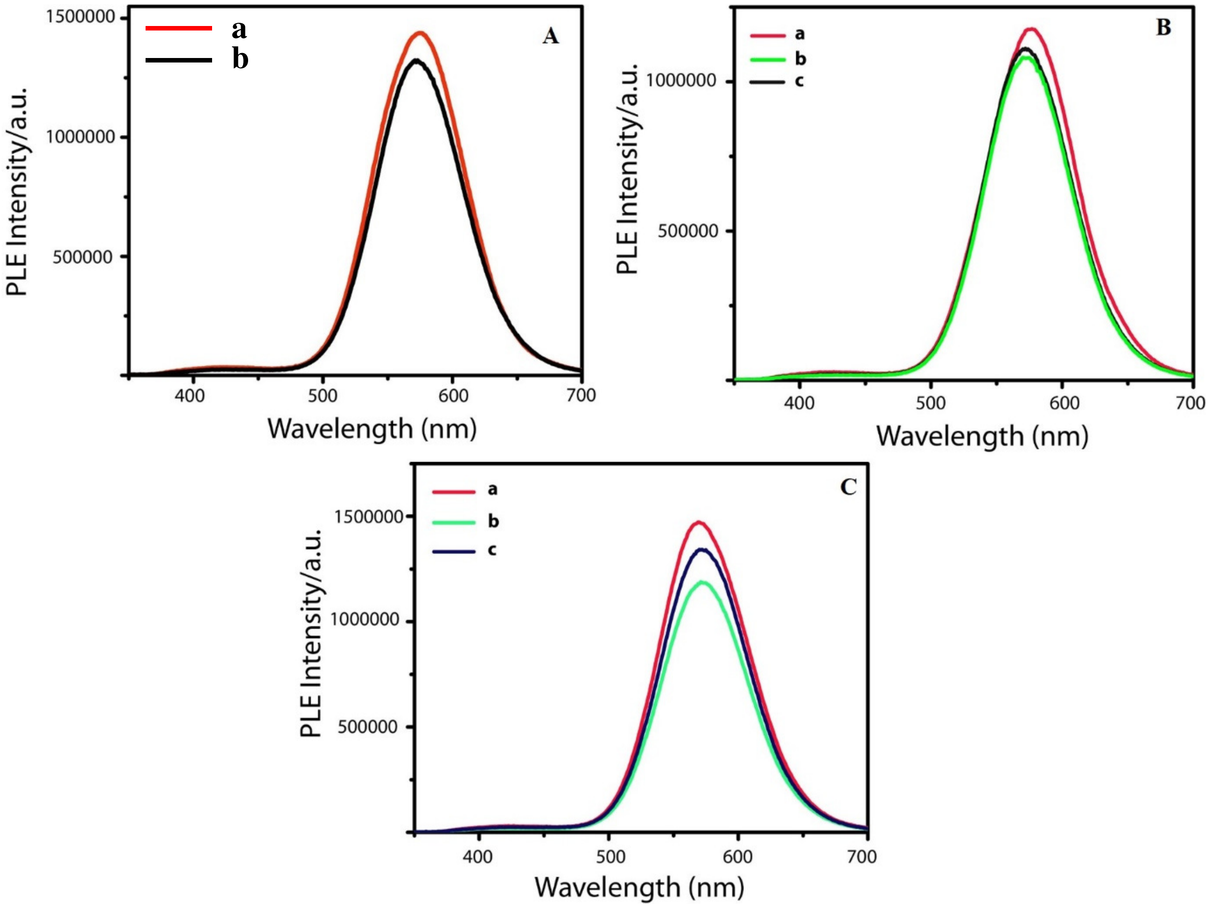

Supplementary Fig. S5 A – C. Effects of addition of (A) Cu^{2+} ions and bilirubin mixture (Cu-BR); PLE spectra of (a) Au nanoclusters and Au nanoclusters treated with (c) Cu-BR, (B) water; PLE spectra of (a) Au nanoclusters, (b) Cu^{2+} and (b) Au nanoclusters treated with water and (C) Cu^{2+} ions followed by bilirubin (BR) on the luminescence of Au nanoclusters (Au NCs) dispersions; PLE spectra of (a) Au nanoclusters, Au nanoclusters treated with (b) Cu^{2+} and then (c) BR. BR means bilirubin.

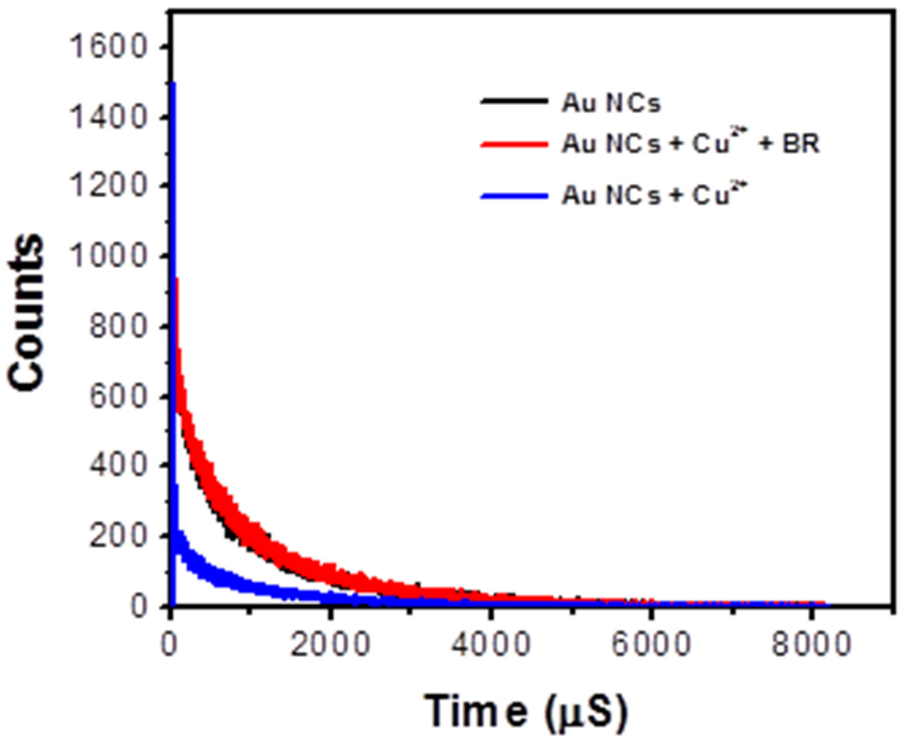

Supplementary Fig. S6. Time-resolved luminescence spectra of Au nanocluster (Au NC) dispersion, Cu^{2+} ion treated nanocluster dispersion and that of Cu^{2+} ion treated nanocluster dispersion following treatment with bilirubin (BR).

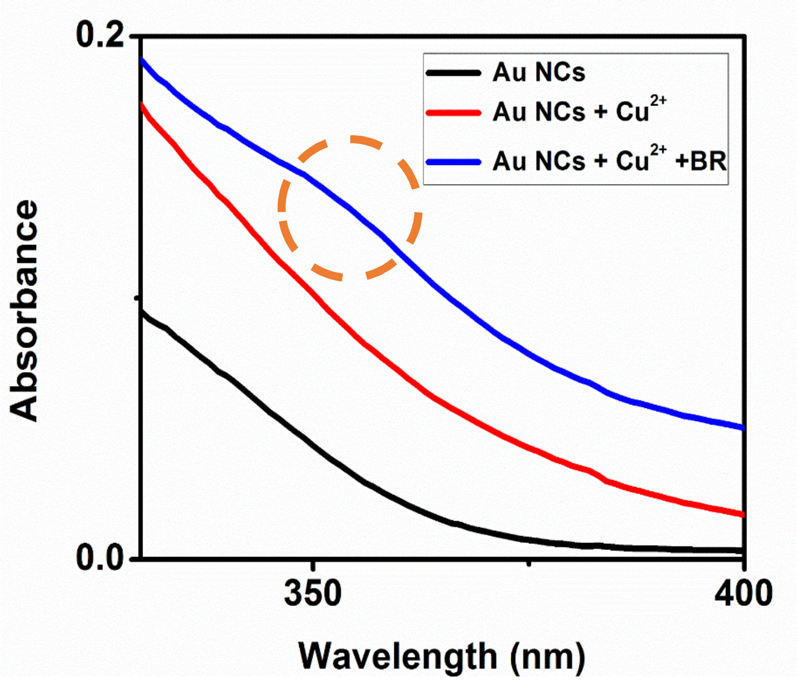

Supplementary Fig. S7. UV-Vis spectra of as-synthesized Au nanocluster (Au NC) dispersion, Cu^{2+} ion treated nanocluster dispersion and that of Cu^{2+} ion treated nanocluster dispersion following treatment with bilirubin (BR).

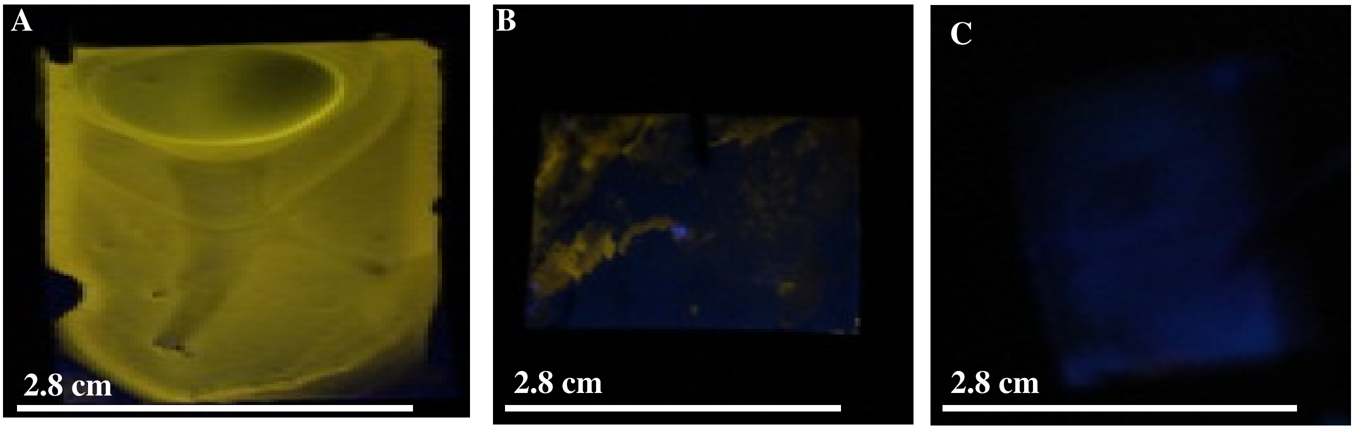

Supplementary Fig. S8 (A) Photograph of the PVDF membrane coated with Au nanoclusters, (B) the same membrane after addition of copper salt showing quenching of luminescence, (C) the film in (B) after addition of water showing no recovery of luminescence intensity. All the photographs were recorded using 254 nm UV light excitation. Dimension of the films was ~ 2.8×2.6 cm^2.

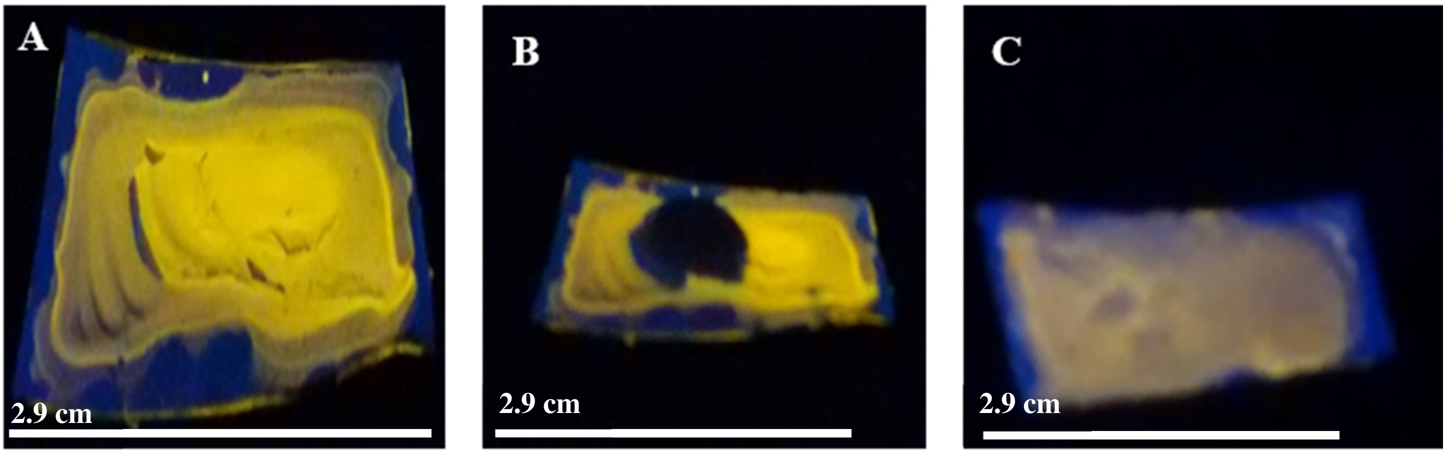

Supplementary Fig. S9. (A) Photograph of Au nanocluster containing PVDF membrane (with the dimension of the films being 2.9 × 1.9 cm^2). (B) Copper salt treated Au nanocluster containing PVDF membrane. The low luminescence region is due to quenching by Cu^{2+} ions added to the membrane. (C) The same film after thumb impression of a jaundice patient. The photographs were recorded following illumination with UV light (254 nm).

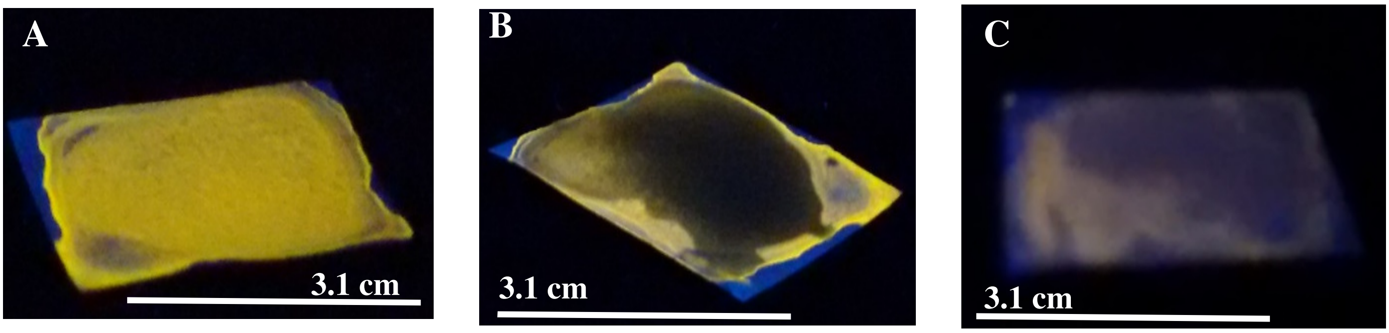

Supplementary Fig. S10. (A) Photograph of Au nanocluster coated PVDF membrane. (B) Copper salt treated Au nanocluster containing film. The low luminescence region is due to quenching by Cu^{2+} ions added to the membrane. (C) The film (shown in (B)) after thumb impression of the same jaundice patient as in figure S9, immediately following thumb imprint on film shown in figure S9. The photographs were recorded following illumination with UV light (254 nm). Dimension of the films was 3.1×2.2 cm^2.

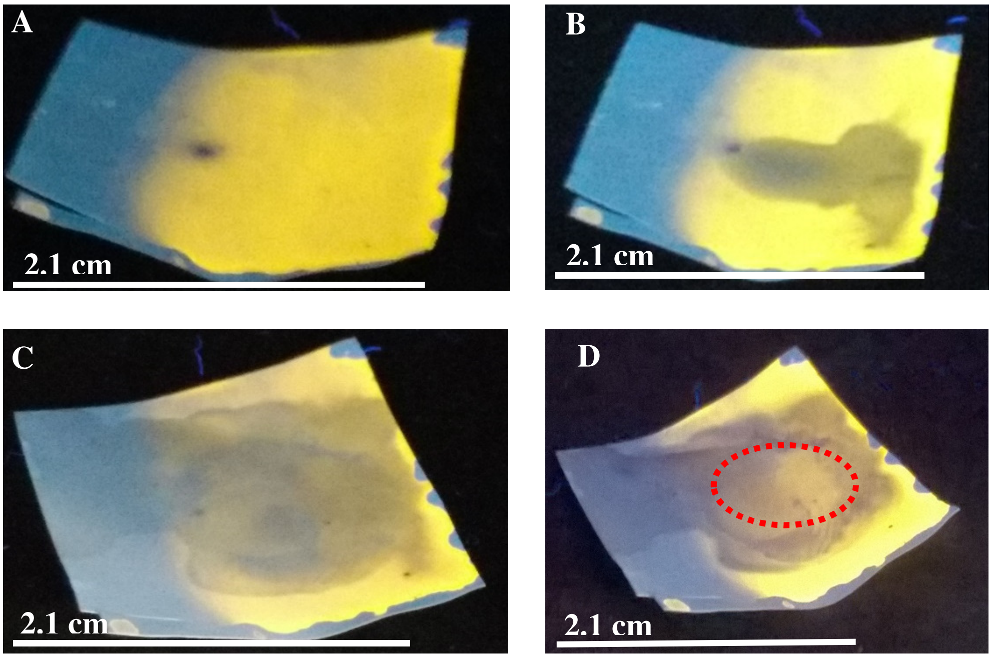

Supplementary Fig. S11. (A) Digital photograph of PVDF membrane coated with gold nanoclusters. (B) Photograph of Cu^{2+} ion treated Au nanoclusters containing PVDF membrane. (C) The same film after thumb impression of a volunteer not affected with hyperbilirubinemia. (D) The film in (C) after addition of bilirubin (1.4×10^{-2} mg/mL). All the photographs were recorded following illumination with UV light (254 nm). Dimension of the films was 2.1×2.5 cm^2.

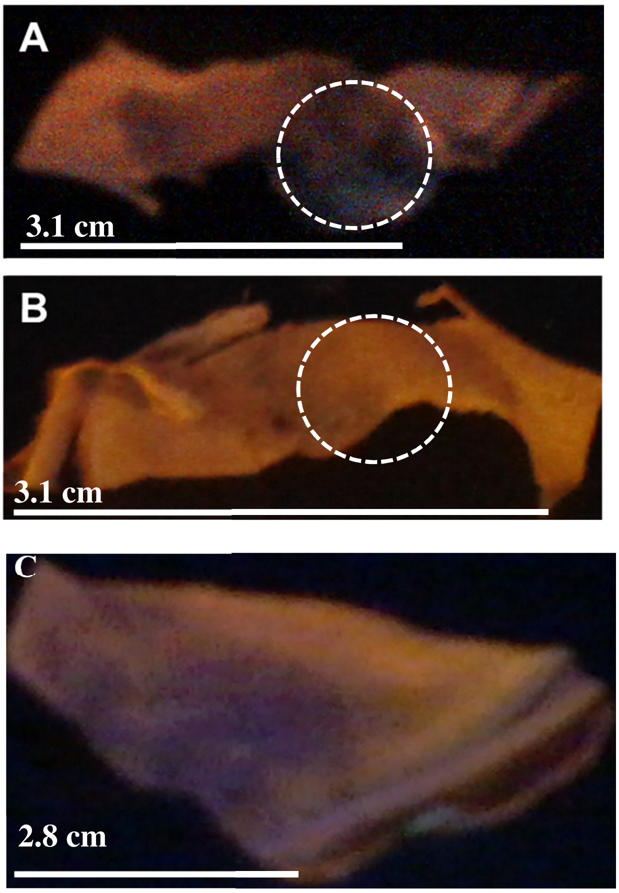

Supplementary Fig. S12. (A) Photograph of copper salt treated Au nanocluster containing film. The low luminescence region is due to quenching by Cu^{2+} ions added to film. (B) The same film after thumb impression of a jaundice patient. The photographs were recorded following illumination with UV light (254 nm). (C) Photograph of copper salt treated Au nanocluster film following thumb impression of a volunteer not affected with hyperbilirubinemia. The photograph was recorded following illumination with UV light (254 nm).

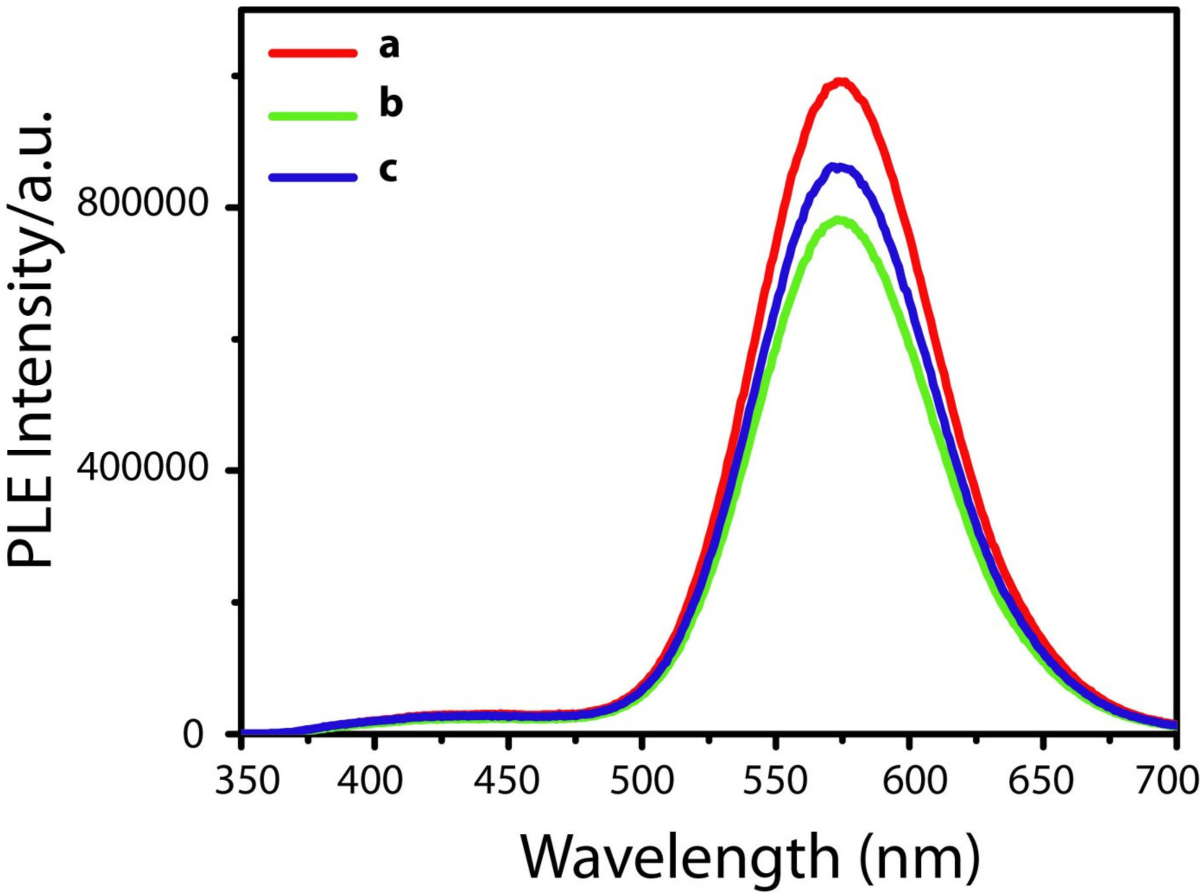

Supplementary Fig. S13 Quenching of luminescence intensity due to Au nanoclusters (Au NCs) in the presence of Cu^{2+} ions followed by recovery of the same upon addition of blood serum containing bilirubin (BR) exceeding normal range. PLE spectra of (a) Au nanoclusters, and following treatment with (b) Cu^{2+} and (c) serum BR exceeding normal range.

Additional figure:

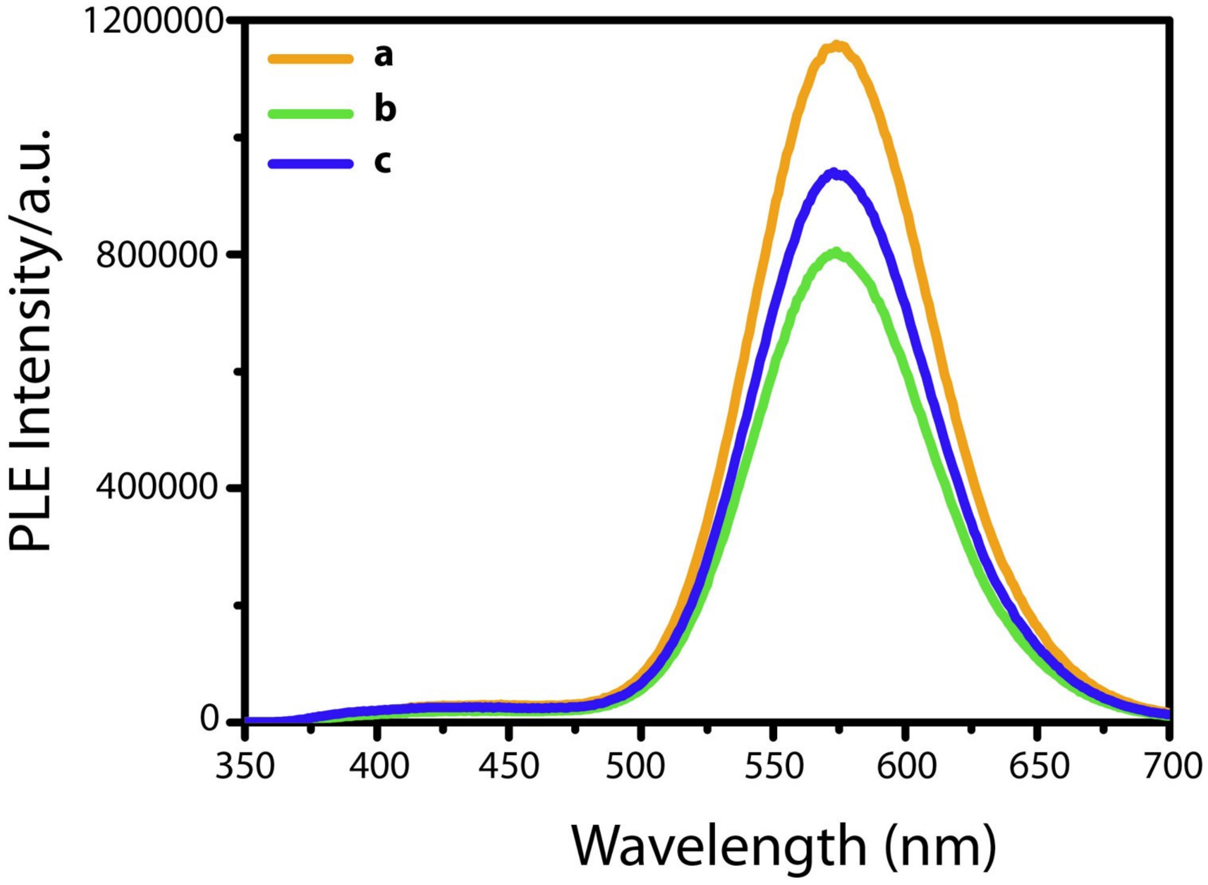

Supplementary Fig. S14 Quenching of luminescence intensity due to Au nanoclusters in the presence of Cu^{2+} ions followed by recovery of the same upon addition of bilirubin (BR). PLE spectra of (a) Au nanoclusters and following addition of (b) Cu^{2+} and (C) BR.

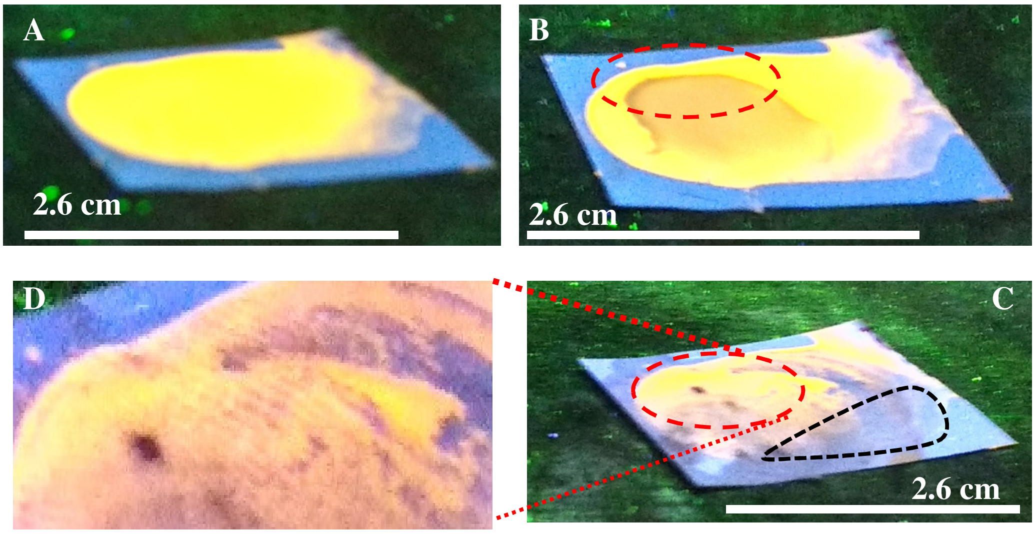

Supplementary Fig. S15. (A) Digital photograph of Au nanoclusters containing PVDF membrane (dimension of the films were 2.6 × 2.1 cm^2). (B) Copper salt (20 μL 12.4 mg/mL) treated Au nanoclusters containing PVDF membrane. The low luminescence region is due to quenching by Cu^{2+} ions added to the membrane. (C) The same film after thumb impression of a jaundice afflicted patient. The photographs were recorded following illumination with UV light (254 nm). (D) Magnified view of the portion of the film in (C) highlighted in red serrated line showing the ridges of the finger of the jaundice afflicted patient causing luminescence recovery of otherwise quenched Au nanoclusters containing film. The part of the film in (C) highlighted in black serrated line showing the blue region is due to shedding a part of the cluster film owing to excess pressure of the thumb during the test.

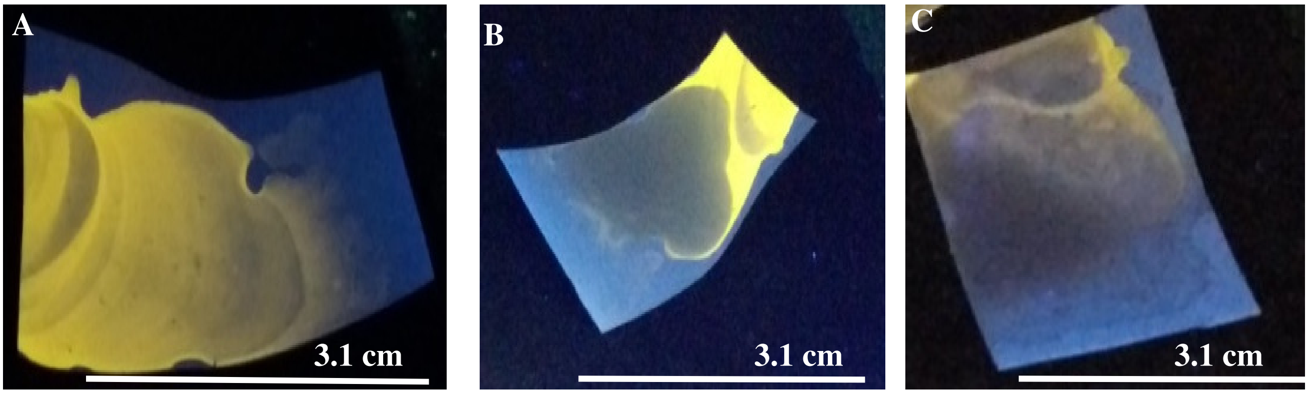

Supplementary Fig. S16. (A) Digital photograph of Au nanoclusters containing PVDF membrane. (B) Copper salt (200 μL 12.4 mg/mL) treated Au nanoclusters containing PVDF membrane. The low luminescence region is due to quenching by Cu^{2+} ions added to the membrane. (C) The same film after thumb impression of a jaundice patient. The photographs were recorded following illumination with UV light (254 nm). The dimension of the films was 3.1×2.2 cm^2. Luminescence intensity quenched in presence of Cu^{2+} did not recover upon acquisition of thumb imprint of a jaundice patient possibly owing to larger amount of Cu^{2+} used.

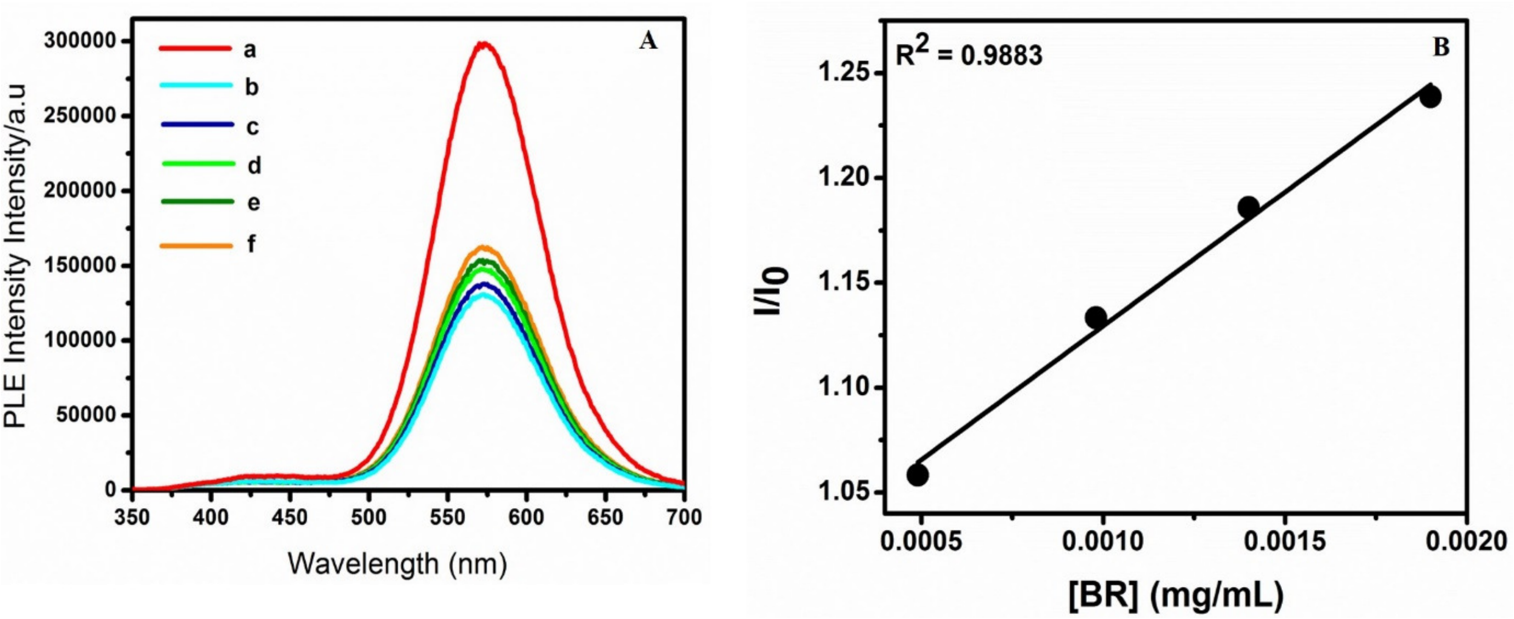

Supplementary Fig. S17. (A) Graphs showing gradual luminescence intensity recovery of Au nanoclusters otherwise quenched in the presence of Cu^{2+} ions. PLE spectra of (a) Au nanocluster dispersion and of that following addition of (b) 4.9×10^{-3} mg/mL Cu^{2+}, (c) 4.9×10^{-4} mg/mL BR, (d) 9.8×10^{-4} mg/mL, (e) 1.4×10^{-3} mg/mL BR and (f) 1.9×10^{-3} mg/mL BR, respectively. (B) Gradual increase of normalized luminescence intensity of the Cu^{2+} quenched Au nanocluster dispersion after addition of bilirubin. I_0 is the reduced luminescence intensity of the nanoclusters in the presence of copper ions. I is the recovered luminescence intensity on subsequent addition of BR. BR means bilirubin.

Explanation of difference in trend of luminescence recovery of quenched gold nanoclusters (in presence of copper ions) as a function of bilirubin concentration

Variation of fluorescence intensity of a fluorophore as a function of concentration is given as:

$$I_F\ (\lambda_E,\lambda_F) = kF_\lambda(\lambda_F)I_0(\lambda_E)\{1-\exp[-2.3\varepsilon(\lambda_E)lc]\}\ \textbf{(1)}$$

I_F = Fluorescence Intensity; λ_E = Excitation wavelength; λ_F = Wavelength at which fluorescence intensity is measured; k = proportionality factor; $F_\lambda(\lambda_F)$ = represents emission spectrum; I_0 = Intensity of incident light; l = optical path length of the sample and c = concentration of sample.

When concentration of fluorophore is less,

Equation 1 can be written as:

$$I_F\ (\lambda_E,\lambda_F) = kF_\lambda(\lambda_F)I_0(\lambda_E)\{2.3\varepsilon(\lambda_E)lc\}\ \textbf{(2)}$$

As per equation (1), fluorescence intensity of a fluorophore should vary exponentially as function of concentration. However, at lower concentration, fluorescence intensity of a fluorophore varies linearly with concentration (in accordance with equation 2).

In Fig. 2B (Manuscript), relatively high of concentration of bilirubin has been used as a consequence of which luminescence intensity has varied exponentially as a function of concentration of bilirubin. On the other hand, upon performing similar experiment, with relatively lower concentration of bilirubin, linear variation of luminescence intensity as a function of bilirubin concentration has been observed (**Fig S17**).

Table S1: The Variation in amount of bilirubin detected by tuning the concentration of Cu^{2+} ions.

Serial No.	Copper ion concentration	Bilirubin concentration	**Reference figure in manuscript (MS) and supporting information (SI)**
**1.**	**5.006×10^{-1} mg/mL**	**8.3×10^{-6} mg/mL**	**Additional figure (SI)**
**2.**	**1.38×10^{-2} mg/mL**	**6.4×10^{-5} mg/mL**	**Figure S13 (SI)**
**3.**	**1.34 mg/mL**	**5×10^{-5}mg/mL**	**Figure S5 C (SI)**
**4.**	**1.38 mg/mL**	**5.8×10^{-4} mg/mL**	**Figure 1 C (MS)**
**5.**	**4.77 mg/mL**	**2.1×10^{-3} mg/mL**	**Figure 2 A (MS)**

Table S2: Details of composition of Au nanoclusters dispersion used for experiments

**Composition of Au nanocluster dispersion**	
**Volume of Au nanoclusters**	**0.1 mL**
**Volume of Glycine buffer**	**0.7 mL**
**Overall pH of the medium**	**< 2.5**

Table S3: Details of the experimental conditions

**Experimental conditions**	
**Temperature**	
**Temperature at which experiments were performed**	**Room temperature**
**Temperature at which Au nanocluster film was dried in oven**	**55˚C**
**Incubation Time**	
**Time taken for synthesis of Au nanoclusters**	**30 min**
**Time taken to dry the films in oven at 55˚C**	**Till the films were completely dry**
**Time taken to dry the copper salt treated Au nanocluster film**	**30 min**
**Duration of collection of thumb print of jaundice patient**	**5 min**

Table S4: Details of patient analysed for thumb imprint based detection of hyperbilirubinemia

SL No.	Response of the test	Total Bilirubin count (mg/dL)
**Patient 1 on day 1**	**Positive**	**6.2**
**Patient 1 on day 2**	**Positive**	**6.2**
**Patient 2**	**Positive**	**4.1**
**Patient 3**	**Positive**	**3.1**
**Patient 4**	**Positive**	**5.2 (approx.)**
**Patient 5**	**Positive**	**37.2**
